# Supplementary material for: Pesticide dynamics in three small agricultural creeks in Hesse, Germany
Source: PeerJ. 2023 Jul 18;11:e15650. doi: 10.7717/peerj.15650 (PMC10361075; doi:10.7717/peerj.15650)
Supplement: Table S1 [file peerj-11-15650-s001.docx]

| **Creek** | **Length [km]** | **Catchment area [km²]** | **Discharge [l/s]** | **coordinates of the event-driven sampler (Decimal degree North; East)** |
| --- | --- | --- | --- | --- |
| Langder Flutgraben | 5.4 | 25.8 | < 100 | 50,460163; 8,934994 |
| Waschbach | 8.3 | 26.4 | < 100 | 50,41038; 8,890606 |
| Weidgraben | 7.4 | -^1)^ | < 100 | 50,403929; 8,910444 |

^1)^ no data available
