# Supplementary material for: Pesticide dynamics in three small agricultural creeks in Hesse, Germany
Source: PeerJ. 2023 Jul 18;11:e15650. doi: 10.7717/peerj.15650 (PMC10361075; doi:10.7717/peerj.15650)
Supplement: Table S2 [file peerj-11-15650-s002.docx]

| **Sampling cycle section** | **1** | **2** | **3** |
| --- | --- | --- | --- |
| **Single sample number** | 1 – 9 | 10 - 18 | 19 - 24 |
| **Time interval between random samples [min]** | 5 | 15 | 30 |
| **Time period per IS [min]** | 20 | 60 | 120 |
| **CS** | I-III | IV-VI | VII, VIII |
