# Supplementary material for: Pesticide dynamics in three small agricultural creeks in Hesse, Germany
Source: PeerJ. 2023 Jul 18;11:e15650. doi: 10.7717/peerj.15650 (PMC10361075; doi:10.7717/peerj.15650)
Supplement: Table S3 [file peerj-11-15650-s003.docx]

|  | **Langder Flugraben** | | | **Waschbach** | | | **Weidgraben** | | |
| --- | --- | --- | --- | --- | --- | --- | --- | --- | --- |
| **Sampling Date** | S | A | E | S | A | E | S | A | E |
| May 20, 2017 | - | - | - | 24^1)^ | 24^1)^ | 0 | - | - | - |
| May 31, 2017 | - | - | - | 22^1)^ | 22^1)^ | 0 | - | - | - |
| June 4, 2017 | - | - | - | 24^1)^ | 24^1)^ | 0 | - | - | - |
| September 18, 2017 | - | - | - | 8 | 8 | 0 | 8 | 8 | 0 |
| October 4, 2017 | - | - | - | 8 | 8 | 0 | 8 | 8 | 0 |
| November 8, 2017 | - | - | - | 7 | 7 | 0 | 8 | 8 | 0 |
| March 30, 2018 | 8 | 8 | 0 | 8 | 8 | 0 | - | - | - |
| May 15, 2018 | 8 | 8 | 8 | 8 | 8 | 8 | 8 | 8 | 2 |
| May 24, 2018 | 8 | 7 | 8 | 8 | 8 | 8 | 8 | 0 | 2 |
| May 31, 2018 | 7 | 6 | 7 | - | - | - | 3 | 0 | 3 |
| June 12/14, 2018 | 8 | 8 | 3 | 8 | 8 | 3 | 8 | 8 | 3 |
| July 24, 2018 | - | - | - | - | - | - | 8 | 0 | 3 |
| August 15, 2018 | - | - | - | - | - | - | 3 | 0 | 0 |

^1)^ At the sampling event no CSs were built, all single samples were analysed.
