# Supplementary material for: Pesticide dynamics in three small agricultural creeks in Hesse, Germany
Source: PeerJ. 2023 Jul 18;11:e15650. doi: 10.7717/peerj.15650 (PMC10361075; doi:10.7717/peerj.15650)
Supplement: Table S4 [file peerj-11-15650-s004.docx]

| **Herbicides** | **Fungicides** | **Insecticides** | **Metabolites** |
| --- | --- | --- | --- |
| Aclonifen | Carbendazim | Acetamiprid | Dimetachlor-ESA |
| Chloridazon | Difenoconazol | Clothianidin | Dimetachlor-OA |
| Chlortoluron | Dimethomorph | Imidacloprid | Dimethenamid-ESA |
| Clomazon | Epoxiconazol | Thiacloprid | Dimethenamid-OA |
| Diflufenican | Fenpropimorph | Thiamethoxam | Diuron-desdimethyl |
| Dimethachlor | Prochloraz |  | Diuron-desmethyl |
| Dimethenamid | Propioconazol |  | Flufenacet-ESA |
| Diuron | Tebuconazol |  | Flufenacet-OA |
| Fluazifop | Triadimenol |  | Metamitron-desamino |
| Flufenacet | Irgarol |  | Metazachlor-ESA |
| Flurtamon |  |  | Metazachlor-OA |
| Isoproturon |  |  | Metolachlor-ESA |
| Mecoprop |  |  | Metolachlor-OA |
| Metamitron |  |  | Propioconazol-desthio |
| Metazachlor |  |  | Terbuthylazine-2-Hydroxy |
| (S)-Metolachlor |  |  | Terbuthylazine-desethyl |
| Napropamide |  |  | Bifenox free acid |
| Propyzamide |  |  |  |
| Prosulfocarb |  |  |  |
| Quinmerac |  |  |  |
| Terbuthylazine |  |  |  |
| Terbutryn |  |  |  |
