# Supplementary material for: Pesticide dynamics in three small agricultural creeks in Hesse, Germany
Source: PeerJ. 2023 Jul 18;11:e15650. doi: 10.7717/peerj.15650 (PMC10361075; doi:10.7717/peerj.15650)
Supplement: Table S5 [file peerj-11-15650-s005.docx]

| **Minute** | **Flow rate [µl]** | **% Eluent A (Milli-Q water+ 0.1% acetic acid)** | **% Eluent B (acetonitrile)** |
| --- | --- | --- | --- |
| 0 | 300 | 98 | 2 |
| 1 | 300 | 98 | 2 |
| 2 | 300 | 80 | 20 |
| 16.5 | 300 | 0 | 100 |
| 19 | 300 | 0 | 100 |
| 19.1 | 300 | 98 | 2 |
| 25 | 300 | 98 | 2 |
