# Supplementary material for: Pesticide dynamics in three small agricultural creeks in Hesse, Germany
Source: PeerJ. 2023 Jul 18;11:e15650. doi: 10.7717/peerj.15650 (PMC10361075; doi:10.7717/peerj.15650)
Supplement: Table S6 [file peerj-11-15650-s006.docx]

| **Parameter** | **ESI pos** | **ESI neg** |
| --- | --- | --- |
| Curtain gas [psi] | 45 | 45 |
| Collision gas | 8 | 8 |
| Ion spray voltage [kV] | 4300 | -4500 |
| Temperature [°C] | 500 | 500 |
| Ion source gas 1 [psi] | 40 | 40 |
| Ion source gas 2 [psi] | 45 | 45 |
| Entrance potential [V] | 10 | -10 |
