# Supplementary material for: Pesticide dynamics in three small agricultural creeks in Hesse, Germany
Source: PeerJ. 2023 Jul 18;11:e15650. doi: 10.7717/peerj.15650 (PMC10361075; doi:10.7717/peerj.15650)
Supplement: Table S7 — Overview about the selected mass transitions (Q1: parent ion, Q3: product ion) and the corresponding compound-dependent instrumental parameters DP (declustering potential), CE (collision energy) and CXP (cell exit potential) as well as the retention times (RT) and surrogate standards used for LC-MS/MS analysis. Mass transitions used for quantification are labeled with an “a” (quantifier), those used for confirmation with a “b” (qualifier). [file peerj-11-15650-s007.docx]

| **Substance name** | **Q1** | **Q3** | **DP [V]** | **CE [eV]** | **CXP [V]** | **RT [min]** | **Surrogat standard** |
| --- | --- | --- | --- | --- | --- | --- | --- |
| Acetamiprid a | 223 | 126.1 | 104 | 33 | 15 | 7.73 | Dimethenamid-d3 |
| Acetamiprid b | 223 | 56.1 | 104 | 23 | 7 | 7.73 | Dimethenamid-d3 |
| Aclonifen a | 265 | 248 | 91 | 23 | 10 | 14.13 | Chlortoluron-d6 |
| Aclonifen b | 265 | 218 | 91 | 34 | 10 | 14.12 | Chlortoluron-d6 |
| Bifenox free acid a | 326 | 282 | -42 | -30 | -10 | 14.13 | Mecoprop-d3 |
| Carbendazim a | 192.1 | 160.1 | 61 | 25 | 10 | 5.63 | Carbendazim-d4 |
| Carbendazim b | 192.1 | 132.1 | 61 | 41 | 10 | 5.63 | Carbendazim-d4 |
| Carbendazim-d4 | 196.2 | 164.1 | 70 | 25 | 10 | 5.59 |  |
| Chloridazone a | 222 | 77 | 110 | 54 | 14 | 7.33 | Imidacloprid-d4 |
| Chloridazone b | 222 | 65 | 110 | 55 | 11 | 7.33 | Imidacloprid-d4 |
| Chlortoluron a | 213 | 72 | 100 | 22 | 13 | 10.3 | Chlortoluron-d6 |
| Chlortoluron b | 213 | 46 | 100 | 36 | 11 | 10.3 | Chlortoluron-d6 |
| Chlortoluron-d6 | 219.1 | 78.1 | 103 | 23 | 13 | 10.24 |  |
| Clomazone a | 240.1 | 125 | 100 | 25 | 11 | 11.7 | Dimethenamid-d3 |
| Clomazone b | 240.1 | 89 | 100 | 65 | 19 | 11.7 | Dimethenamid-d3 |
| Clothianidin a | 250 | 132 | 90 | 15 | 10 | 7.1 | Imidacloprid-d4 |
| Clothianidin b | 250 | 169 | 90 | 5 | 10 | 7.1 | Imidacloprid-d4 |
| DCPMU a | 217 | 160 | -40 | -18 | -10 | 10.1 | Diuron-d6 |
| DCPMU b | 219 | 162 | -40 | -18 | -10 | 10.1 | Diuron-d6 |
| DCPU a | 203 | 160 | -50 | -20 | -8 | 9.3 | Diuron-d6 |
| DCPU b | 205 | 162 | -50 | -18 | -12 | 9.3 | Diuron-d6 |
| Desamino-Metamitron a | 188.1 | 104 | 60 | 28 | 10 | 6.6 | Carbendazim-d4 |
| Desamino-Metamitron b | 188.1 | 77 | 60 | 28 | 10 | 6.6 | Carbendazim-d4 |
| Difenoconazole a | 406.1 | 251 | 100 | 36 | 13 | 14.3 | Difenoconazole-d6 |
| Difenoconazole b | 408 | 253 | 100 | 36 | 14 | 14.3 | Difenoconazole-d6 |
| Difenoconazole-d6 | 412 | 251 | 145 | 37 | 14 | 14.3 |  |
| Diflufenican a | 395 | 266 | 118 | 35 | 15 | 15.4 | Diflufenican-d3 |
| Diflufenican b | 395 | 246 | 118 | 48 | 16 | 15.4 | Diflufenican-d3 |
| Diflufenican-d3 | 398 | 268 | 140 | 39 | 13 | 15.4 |  |
| Dimethachlor a | 256.1 | 148.1 | 30 | 34 | 18 | 11.6 | Dimethenamid-d3 |
| Dimethachlor b | 258 | 148 | 30 | 34 | 16 | 11.6 | Dimethenamid-d3 |
| Dimethachlor-ESA a | 300 | 121 | -30 | -27 | -5 | 8.2 | Diuron-d6 |
| Dimethachlor-ESA b | 300 | 80 | -30 | -55 | -9 | 8.2 | Diuron-d6 |
| Dimethachlor-OA a | 250 | 178 | -20 | -14 | -14 | 8.0 | Diuron-d6 |
| Dimethachlor-OA b | 250.1 | 144 | -20 | -35 | -14 | 8.0 | Diuron-d6 |
| Dimethenamid a | 276 | 168 | 20 | 34 | 9 | 12.6 | Dimethenamid-d3 |
| Dimethenamid b | 276 | 111 | 20 | 35 | 14 | 12.6 | Dimethenamid-d3 |
| Dimethenamid-d3 | 279.1 | 111 | 100 | 42 | 13 | 12.5 |  |
| Dimethenamid-ESA a | 320 | 80 | -50 | -68 | -9 | 9.1 | Diuron-d6 |
| Dimethenamid-ESA b | 320 | 120 | -50 | -50 | -13 | 9.1 | Diuron-d6 |
| Dimethenamid-OA a | 272 | 126 | 28 | 40 | 12 | 8.5 | Dimethenamid-d3 |
| Dimethenamid-OA b | 272 | 111 | 28 | 36 | 10 | 8.5 | Dimethenamid-d3 |
| Dimethomorph a | 388.1 | 301.1 | 105 | 28 | 16 | 11.8 | Chlortoluron-d6 |
| Dimethomorph b | 388.1 | 165.1 | 105 | 43 | 15 | 11.8 | Chlortoluron-d6 |
| Diuron a | 231 | 186 | -60 | -25 | -8 | 10.8 | Diuron-d6 |
| Diuron b | 233 | 188 | -60 | -25 | -8 | 10.8 | Diuron-d6 |
| Diuron-d6 | 237 | 186 | -70 | -25 | -9 | 10.7 |  |
| Epoxiconazole a | 330.1 | 121 | 65 | 35 | 6 | 12.7 | Epoxiconazole-d4 |
| Epoxiconazole b | 330.1 | 75 | 65 | 95 | 6 | 12.7 | Epoxiconazole-d4 |
| Epoxiconazole-d4 | 334.1 | 125.3 | 60 | 35 | 10 | 12.7 |  |
| Fenpropimorph a | 304.3 | 147.2 | 81 | 41 | 10 | 9.1 | Tebuconazole-d6 |
| Fenpropimorph b | 304.3 | 117.1 | 81 | 77 | 10 | 9.1 | Tebuconazole-d6 |
| Fluazifop a | 328 | 254 | 110 | 34 | 16 | 12.3 | Fluazifop-d4 |
| Fluazifop b | 328 | 238 | 110 | 43 | 14 | 12.3 | Fluazifop-d4 |
| Fluazifop-d4 | 332 | 238 | 80 | 30 | 11 | 12.3 |  |
| Flufenacet a | 364.1 | 152 | 80 | 16 | 9 | 13.8 | Flufenacet-d4 |
| Flufenacet b | 364.1 | 194 | 80 | 27 | 10 | 13.8 | Flufenacet-d4 |
| Flufenacet-d4 | 368.1 | 128 | 105 | 45 | 20 | 13.8 |  |
| Flufenacet-ESA a | 274 | 80 | -50 | -60 | -13 | 8.5 | Diuron-d6 |
| Flufenacet-ESA b | 274 | 77 | -50 | -43 | -9 | 8.5 | Diuron-d6 |
| Flufenacet-OA a | 224 | 136 | -10 | -29 | -10 | 8.0 | Diuron-d6 |
| Flufenacet-OA b | 224 | 95 | -10 | -36 | -10 | 8.0 | Diuron-d6 |
| Flurtamone a | 334.3 | 178.1 | 160 | 60 | 22 | 12.3 | Flufenacet-d4 |
| Flurtamone b | 334.3 | 247.1 | 160 | 36 | 13 | 12.3 | Flufenacet-d4 |
| Ibuprofen a | 205.1 | 161.0 | -30 | -10 | -11 | 13.3 | Ibuprofen-d3 |
| Ibuprofen a | 205.1 | 159.0 | -30 | -6 | -9 | 13.3 | Ibuprofen-d3 |
| Ibuprofen-d3 | 208.1 | 164.0 | -55 | -10 | -11 | 13.3 |  |
| Imidacloprid a | 256.1 | 209 | 60 | 25 | 4 | 7.4 | Imidacloprid-d4 |
| Imidacloprid b | 256.1 | 175.1 | 60 | 30 | 13 | 7.4 | Imidacloprid-d4 |
| Imidacloprid-d4 | 260.1 | 179.1 | 80 | 25 | 8 | 7.4 |  |
| Irgarol a | 254 | 198 | 70 | 26 | 6 | 12.7 | Irgarol-d9 |
| Irgarol b | 254 | 83 | 70 | 41 | 6 | 12.7 | Irgarol-d9 |
| Irgarol-d9 | 263 | 199 | 40 | 29 | 16 | 12.6 |  |
| Isoproturon a | 207 | 165.1 | 65 | 22 | 14 | 10.7 | Isoproturon-d6 |
| Isoproturon b | 207 | 72 | 65 | 35 | 10 | 10.7 | Isoproturon-d6 |
| Isoproturon-d6 | 213.2 | 78 | 65 | 30 | 10 | 10.7 |  |
| Mecoprop a | 213 | 141 | -35 | -20 | -5 | 11.7 | Mecoprop-d3 |
| Mecoprop b | 215 | 143 | -35 | -20 | -5 | 11.7 | Mecoprop-d3 |
| Mecoprop-d3 | 216 | 144 | -40 | -25 | -10 | 11.6 |  |
| Metamitron a | 203.1 | 104 | 60 | 33 | 8 | 7.1 | Metamitron-d5 |
| Metamitron b | 203.1 | 175.1 | 60 | 23 | 10 | 7.1 | Metamitron-d5 |
| Metamitron-d5 | 208.1 | 180.1 | 60 | 23 | 10 | 7.0 |  |
| Metazachlor a | 278.1 | 134.1 | 35 | 30 | 10 | 11.4 | Metazachlor-d6 |
| Metazachlor b | 278.1 | 210 | 35 | 15 | 10 | 11.4 | Metazachlor-d6 |
| Metazachlor-d6 | 284.1 | 140.1 | 45 | 30 | 10 | 11.4 |  |
| Metazachlor-ESA a | 324.1 | 69.1 | 24 | 15 | 7 | 8.0 | Chlortoluron-d6 |
| Metazachlor-ESA b | 324.1 | 134.1 | 24 | 34 | 7 | 8.0 | Chlortoluron-d6 |
| Metazachlor-OA a | 274.1 | 134.1 | 27 | 28 | 21 | 7.5 | Metazachlor-d6 |
| Metazachlor-OA b | 274.1 | 105.1 | 27 | 49 | 16 | 7.5 | Metazachlor-d6 |
| Metolachlor a | 284.1 | 252 | 45 | 20 | 5 | 13.7 | Metolachlor-d6 |
| Metolachlor b | 286.1 | 176 | 45 | 35 | 10 | 13.7 | Metolachlor-d6 |
| Metolachlor-d6 | 290.1 | 258.1 | 45 | 20 | 5 | 13.7 |  |
| Metolachlor-ESA a | 328 | 80 | -50 | -70 | -10 | 11.5 | Diuron-d6 |
| Metolachlor-ESA b | 328 | 121 | -50 | -31 | -10 | 11.5 | Diuron-d6 |
| Metolachlor-OA a | 278 | 206 | -10 | -15 | -10 | 10.7 | Diuron-d6 |
| Metolachlor-OA b | 278 | 158 | -10 | -27 | -10 | 10.7 | Diuron-d6 |
| Napropamide a | 272 | 128 | 128 | 55 | 20 | 13.2 | - |
| Napropamide b | 272 | 153 | 128 | 43 | 21 | 13.2 | - |
| Prochloraz a | 376 | 308 | 120 | 32 | 18 | 13.4 | Prochloraz-d7 |
| Prochloraz b | 376 | 70 | 120 | 60 | 12 | 13.4 | Prochloraz-d7 |
| Prochloraz-d7 | 383 | 266 | 124 | 56 | 12 | 13.4 |  |
| Propiconazole a | 342.1 | 159 | 76 | 45 | 10 | 13.8 | Propioconazole-d5 |
| Propiconazole b | 344.1 | 161 | 76 | 37 | 10 | 13.8 | Propioconazole-d5 |
| Propiconazole-d5 | 347.2 | 159.1 | 80 | 34 | 10 | 13.8 |  |
| Propyzamide a | 254 | 228 | -56 | -20 | -5 | 13.0 | Propyzamide-d3 |
| Propyzamide b | 254 | 145 | -56 | -20 | -5 | 13.0 | Propyzamide-d3 |
| Propyzamide-d3 | 257 | 148 | -56 | -20 | -5 | 13.0 |  |
| Prosulfocarb a | 252.1 | 91 | 90 | 36 | 16 | 13.7 | Carbendazim-d4 |
| Prosulfocarb b | 252.1 | 65 | 90 | 75 | 15 | 13.7 | Carbendazim-d4 |
| Prothioconazole-desthio a | 312.1 | 70.1 | 50 | 60 | 12 | 12.6 | Propioconazole-d5 |
| Prothioconazole-desthio b | 312.1 | 125.1 | 50 | 45 | 11 | 12.6 | Propioconazole-d5 |
| Quinmerac a | 222 | 141 | 50 | 45 | 18 | 7.3 | Quinmerac-d4 |
| Quinmerac b | 222 | 149 | 50 | 46 | 15 | 7.3 | Quinmerac-d4 |
| Quinmerac-d4 | 226 | 145 | 60 | 46 | 16 | 7.3 |  |
| Tebuconazole a | 308.1 | 70 | 81 | 49 | 11 | 13.1 | Tebuconazole-d6 |
| Tebuconazole b | 310.1 | 70 | 81 | 45 | 11 | 13.1 | Tebuconazole-d6 |
| Tebuconazole-d6 | 314.3 | 72.1 | 84 | 59 | 10 | 13.1 |  |
| Terbuthylazine a | 230.1 | 174.1 | 61 | 25 | 40 | 12.3 | Terbutylazine-d5 |
| Terbuthylazine b | 230.1 | 104 | 61 | 45 | 11 | 12.3 | Terbutylazine-d5 |
| Terbuthylazine-2-Hydroxy a | 212.2 | 156.1 | 70 | 18 | 8 | 5.9 | Terbutylazine-d5 |
| Terbuthylazine-2-Hydroxy b | 212.2 | 97 | 70 | 38 | 8 | 5.9 | Terbutylazine-d5 |
| Terbuthylazine-d5 | 235.2 | 104 | 61 | 45 | 11 | 12.2 |  |
| Terbuthylazine-desethyl a | 202.1 | 146.1 | 60 | 20 | 8 | 9.5 | Terbutylazin-desethyl-d9 |
| Terbuthylazine-desethyl b | 202.1 | 104 | 60 | 37 | 8 | 9.5 | Terbutylazin-desethyl-d9 |
| Terbuthylazine-desethyl-d9 | 211.1 | 104 | 60 | 40 | 8 | 9.4 |  |
| Terbutryn a | 242 | 186 | 50 | 25 | 15 | 12.4 | Terbutryn-d5 |
| Terbutryn b | 242 | 91 | 50 | 38 | 11 | 12.4 | Terbutryn-d5 |
| Terbutryn-d5 | 247 | 191 | 50 | 25 | 15 | 12.4 |  |
| Thiacloprid a | 253 | 126 | 116 | 29 | 8 | 8.6 | Dimethenamid-d3 |
| Thiacloprid b | 255 | 128 | 116 | 29 | 8 | 8.6 | Dimethenamid-d3 |
| Thiamethoxam a | 292 | 211 | 71 | 17 | 8 | 6.5 | - |
| Thiamethoxam b | 292 | 181 | 71 | 27 | 12 | 6.5 | - |
| Triadimenol a | 296.1 | 70 | 60 | 37 | 12 | 11.8 | Triadimenol-d4 |
| Triadimenol b | 298 | 70 | 60 | 38 | 8 | 11.8 | Triadimenol-d4 |
| Triadimenol-d4 | 300.1 | 70 | 23 | 38 | 8 | 11.8 |  |
