# Supplementary material for: Pesticide dynamics in three small agricultural creeks in Hesse, Germany
Source: PeerJ. 2023 Jul 18;11:e15650. doi: 10.7717/peerj.15650 (PMC10361075; doi:10.7717/peerj.15650)
Supplement: Table S8 [file peerj-11-15650-s008.docx]

Table S8: Acute EC/LC_50_ values for the most sensitive taxon for each compound obtained from the Pesticide Properties DataBase (PPDB) operated by the University of Hertfordshire (http://sitem.herts.ac.uk/aeru/iupac/index.htm).

| **Substance** | **Test^1^** | **Value [ng/L]^1^** |
| --- | --- | --- |
| Acetamiprid | EC_50_ (48h) *D. magna* | 49800000 |
|  | LC_50_ (96h) *C. riparius* | 2800000 |
|  | EC_50_ (7d) *L. gibba* | 1000000 |
|  | EC_50_ (72h) *S. subspicatus* | 98300000 |
|  | LC_50_ (96h) *O. mykiss* | 100000000 |
| Aclonifen | EC_50_ (48h) *D. magna* | 1200000 |
|  | LC_50_ (96h) *C. riparius* | -^2^ |
|  | EC_50_ (7d) *L. gibba* | 6000 |
|  | EC_50_ (72h) *N. pelliculosa* | 470000 |
|  | LC_50_ (96h) *O. mykiss* | 670000 |
| Bifenox free acid | EC_50_ (48h) *D. magna* | 660000 |
|  | LC_50_ (96h) *C. riparius* | -^2^ |
|  | EC_50_ (7d) *L. gibba* | 2100 |
|  | EC_50_ (72h) *N. pelliculosa* | 180 |
|  | LC_50_ (96h) *O. mykiss* | 670000 |
| Carbendazim | EC_50_ (48h) *D. magna* | 150000 |
|  | LC_50_ (96h) *C. riparius* | -^2^ |
|  | EC_50_ (7d) *L. gibba* | -^2^ |
|  | EC_50_ (72h) *S. subspicatus* | 7700000 |
|  | LC_50_ (96h) *O. mykiss* | 190000 |
| Clomazon | EC_50_ (48h) *D. magna* | 12700000 |
|  | LC_50_ (96h) *C. riparius* | -^2^ |
|  | EC_50_ (7d) *L. gibba* | 34000000 |
|  | EC_50_ (72h) *N. pelliculosa* | 136000 |
|  | LC_50_ (96h) *O. mykiss* | 14400000 |
| Chloridazon | EC_50_ (48h) *D. magna* | 132000000 |
|  | LC_50_ (96h) *C. riparius* | -^2^ |
|  | EC_50_ (7d) *L. gibba* | 3160000 |
|  | EC_50_ (72h) *unknown species* | 730000 |
|  | LC_50_ (96h) *O. mykiss* | 41300000 |
| Chlortoluron | EC_50_ (48h) *D. magna* | 67000000 |
|  | LC_50_ (96h) *C. riparius* | -^2^ |
|  | EC_50_ (7d) *L. gibba* | 38000 |
|  | EC_50_ (72h) *P. subcapitata* | 82000 |
|  | LC_50_ (96h) *O. mykiss* | 7700000 |
| Clothianidin | EC_50_ (48h) *D. magna* | 40000000 |
|  | LC_50_ (96h) *C. riparius* | 29000 |
|  | EC_50_ (7d) *L. gibba* | 121000000 |
|  | EC_50_ (72h) *P. subcapitata* | 55000000 |
|  | LC_50_ (96h) *O. mykiss* | 104200000 |
| DCPMU | EC_50_ (48h) *D.magna* | 49000000 |
|  | LC_50_ (96h) *C. riparius* | -^2^ |
|  | EC_50_ (7d) *L. gibba* | -^2^ |
|  | EC_50_ (72h) *D. subspicatus* | 13000 |
|  | LC_50_ (96h) *O. mykiss* | -^2^ |
| DCPU | EC_50_ (48h) *D. magna* | 14000000 |
|  | LC_50_ (96h) *C. riparius* | -^2^ |
|  | EC_50_ (7d) *L. gibba* | -^2^ |
|  | EC_50_ (72h)  *D. subspicatus* | 5290000 |
|  | LC_50_ (96h) *O. mykiss* | -^2^ |
| Metamitron-desamino | EC_50_ (48h) *D. magna* | 745000000 |
|  | LC_50_ (96h) *C. riparius* | 100000000 |
|  | EC_50_ (7d) *L. gibba* | 73200000 |
|  | EC_50_ (72h) *P. subcapitata* | 73500000 |
|  | LC_50_ (96h) *O. mykiss* | 1000000000 |
| Difenoconazol | EC_50_ (48h) *D. magna* | 770000 |
|  | LC_50_ (96h) *C. riparius* | 770000 |
|  | EC_50_ (7d) *unknown species* | 2500000 |
|  | EC_50_ (72h) *S. subspicatus* | 32000 |
|  | LC_50_ (96h) *O. mykiss* | 1100000 |
| Diflufenican | EC_50_ (48h) *D. magna* | 240000 |
|  | LC_50_ (96h) *C. riparius* | -^2^ |
|  | EC_50_ (7d) *L. gibba* | 56000 |
|  | EC_50_ (72h) *S. subspicatus* | 250 |
|  | LC_50_ (96h) *O. mykiss* | 99000 |
| Dimethachlor | EC_50_ (48h) *D. magna* | 24000000 |
|  | LC_50_ (96h) *C. riparius* | -^2^ |
|  | EC_50_ (7d) *L. gibba* | 35000 |
|  | EC_50_ (72h) *P. subcapitata* | 6500 |
|  | LC_50_ (96h) *O. mykiss* | 3900000 |
| Dimethachlor-ESA | EC_50_ (48h) *D.magna* | -^2^ |
|  | LC_50_ (96h) *C. riparius* | -^2^ |
|  | EC_50_ (7d) *L. gibba* | -^2^ |
|  | EC_50_ (72h) *S. subspicatus* | -^2^ |
|  | LC_50_ (96h) *O. mykiss* | -^2^ |
| Dimethachlor-OA | EC_50_ (48h) *D. magna* | -^2^ |
|  | LC_50_ (96h) *C. riparius* | -^2^ |
|  | EC_50_ (7d) *L. gibba* | -^2^ |
|  | EC_50_ (72h) *S. subspicatus* | -^2^ |
|  | LC_50_ (96h) *O. mykiss* | -^2^ |
| Dimethenamid | EC_50_ (48h) *D. magna* | 16000000 |
|  | LC_50_ (96h) *C. riparius* | -^2^ |
|  | EC_50_ (7d) *L. gibba* | 28000 |
|  | EC_50_ (72h) *S. subspicatus* | 62000 |
|  | LC_50_ (96h) *O. mykiss* | 2600000 |
| Dimethenamid-ESA | EC_50_ (48h) *D. magna* | 100000000 |
|  | LC_50_ (96h) *C. riparius* | -^2^ |
|  | EC_50_ (7d) *L. gibba* | -^2^ |
|  | EC_50_ (72h) *P. subcapitata* | 208000000 |
|  | LC_50_ (96h) *O. mykiss* | 100000000 |
| Dimethenamid-OA | EC_50_ (48h) *D. magna* | 95000000 |
|  | LC_50_ (96h) *C. riparius* | -^2^ |
|  | EC_50_ (7d) *L. gibba* | -^2^ |
|  | EC_50_ (72h) *R. subcapitata* | 94000000 |
|  | LC_50_ (96h) *O. mykiss* | 87000000 |
| Dimethomorph | EC_50_ (48h) *D. magna* | 20100000 |
|  | LC_50_ (96h) *C. riparius* | -^2^ |
|  | EC_50_ (7d) *L. gibba* | 1000000 |
|  | EC_50_ (72h) *S. subspicatus* | 29200000 |
|  | LC_50_ (96h) *O. mykiss* | 6100000 |
| Diuron | EC_50_ (48h) *D. magna* | 5700000 |
|  | LC_50_ (96h) *C. riparius* | -^2^ |
|  | EC_50_ (7d) *L. gibba* | 18300 |
|  | EC_50_ (72h) *S. quadricauda* | 2700 |
|  | LC_50_ (96h) *O. mykiss* | 6700000 |
| Epoxiconazol | EC_50_ (48h) *D. magna* | 3130000 |
|  | LC_50_ (96h) *C. riparius* | 62500 |
|  | EC_50_ (7d) *L. gibba* | 14000 |
|  | EC_50_ (72h) *P. subcapitata* | 10690000 |
|  | LC_50_ (96h) *O. mykiss* | 920000 |
| Fenpropimorph | EC_50_ (48h) *D. magna* | 2240000 |
|  | LC_50_ (96h) *C. riparius* | -^2^ |
|  | EC_50_ (7d) *L. gibba* | -^2^ |
|  | EC_50_ (72h) *P. subcapitata* | 327000 |
|  | LC_50_ (96h) *L. macrochirus* | 2300000 |
| Fluazifop | EC_50_ (48h) *D. magna* | -^2^ |
|  | LC_50_ (96h) *C. riparius* | -^2^ |
|  | EC_50_ (7d) *L. gibba* | -^2^ |
|  | EC_50_ (72h) *S. subspicatus* | -^2^ |
|  | LC_50_ (96h) *O. mykiss* | -^2^ |
| Flufenacet | EC_50_ (48h) *D. magna* | 30900000 |
|  | LC_50_ (96h) *C. riparius* | -^2^ |
|  | EC_50_ (7d) *L. gibba* | 2000 |
|  | EC_50_ (72h) *R. subcapitata* | 2040 |
|  | LC_50_ (96h) *L. macrochirus* | 2130000 |
| Flufenacet-ESA | EC_50_ (48h) *D. magna* | 87900000 |
|  | LC_50_ (96h) *C. riparius* | -^2^ |
|  | EC_50_ (7d) *L. gibba* | 79500000 |
|  | EC_50_ (72h) *S. subspicatus* | 86700000 |
|  | LC_50_ (96h) *O. mykiss* | 86700000 |
| Flufenacet-OA | EC_50_ (48h) *D. magna* | -^2^ |
|  | LC_50_ (96h) *C. riparius* | -^2^ |
|  | EC_50_ (7d) *L. gibba* | 100000000 |
|  | EC_50_ (72h) *P. subcapitata* | 100000000 |
|  | LC_50_ (96h) *O. mykiss* | -^2^ |
| Flurtamon | EC_50_ (48h) *D. magna* | 13000000 |
|  | LC_50_ (96h) *C. riparius* | -^2^ |
|  | EC_50_ (7d) *L. gibba* | 14100 |
|  | EC_50_ (72h) *P. subcapitata* | 73000 |
|  | LC_50_ (96h) *P. promelas* | 6640000 |
| Imidacloprid | EC_50_ (48h) *D. magna* | 85000000 |
|  | LC_50_ (96h) *C. riparius* | 55000 |
|  | EC_50_ (7d) *L. gibba* | -^2^ |
|  | EC_50_ (72h) *S. subspicatus* | 10000000 |
|  | LC_50_ (96h) *O. mykiss* | 83000000 |
| Irgarol | EC_50_ (48h) *D. magna* | 2400000 |
|  | LC_50_ (96h) *C. riparius* | -^2^ |
|  | EC_50_ (7d) *L. gibba* | 11000 |
|  | EC_50_ (72h) *S. capricornutum* | 2300 |
|  | LC_50_ (96h) *O. mykiss* | 860000 |
| Isoproturon | EC_50_ (48h) *D. magna* | 580000 |
|  | LC_50_ (96h) *C. riparius* | -^2^ |
|  | EC_50_ (7d) *L. gibba* | 31000 |
|  | EC_50_ (72h) *N. pelliculosa* | 13000 |
|  | LC_50_ (96h) *unknown species* | 18000000 |
| Mecoprop | EC_50_ (48h) *D. magna* | 200000000 |
|  | LC_50_ (96h) *C. riparius* | -^2^ |
|  | EC_50_ (7d) *unknown species* | 40200000 |
|  | EC_50_ (72h) *unknown species* | 237000000 |
|  | LC_50_ (96h) unknown species | 240000000 |
| Metamitron | EC_50_ (48h) *D. magna* | 5700000 |
|  | LC_50_ (96h) *C. riparius* | -^2^ |
|  | EC_50_ (7d) *L. gibba* | 400000 |
|  | EC_50_ (72h) *P. subcapitata* | 400000 |
|  | LC_50_ (96h) *O. mykiss* | 190000000 |
| Metazachlor | EC_50_ (48h) *D. magna* | 33000000 |
|  | LC_50_ (96h) *C. riparius* | -^2^ |
|  | EC_50_ (7d) *L. gibba* | 2300 |
|  | EC_50_ (72h) *P. subcapitata* | 16200 |
|  | LC_50_ (96h) *O. mykiss* | 8500000 |
| Metazachlor-ESA | EC_50_ (48h) *D. magna* | 93800000 |
|  | LC_50_ (96h) *C. riparius* | -^2^ |
|  | EC_50_ (7d) *L. gibba* | 91900000 |
|  | EC_50_ (72h) *P. subcapitata* | 93800000 |
|  | LC_50_ (96h) *O. mykiss* | 93800000 |
| Metazachlor-OA | EC_50_ (48h) *D. magna* | 100000000 |
|  | LC_50_ (96h) *C. riparius* | -^2^ |
|  | EC_50_ (7d) *L. gibba* | 100000000 |
|  | EC_50_ (72h) *S. subspicatus* | 25700000 |
|  | LC_50_ (96h) *O. mykiss* | 100000000 |
| (S)-Metolachlor | EC_50_ (48h) *D. magna* | 707000 |
|  | LC_50_ (96h) *C. riparius* | -^2^ |
|  | EC_50_ (7d) *L. gibba* | 43000 |
|  | EC_50_ (72h) *P. subcapitata* | 57100000 |
|  | LC_50_ (96h) *O. mykiss* | 3900000 |
| Metolachlor-ESA | EC_50_ (48h) *D. magna* | 100000000 |
|  | LC_50_ (96h) *C. riparius* | -^2^ |
|  | EC_50_ (7d) *L. gibba* | 21700000 |
|  | EC_50_ (72h) *P. subcapitata* | 100000000 |
|  | LC_50_ (96h) *O. mykiss* | 43000000 |
| Metolachlor-OA | EC_50_ (48h) *D. magna* | 16600000 |
|  | LC_50_ (96h) *C. riparius* | -^2^ |
|  | EC_50_ (7d) *L. gibba* | 88600000 |
|  | EC_50_ (72h) *D. subspicatus* | 77600000 |
|  | LC_50_ (96h) *O. mykiss* | 100000000 |
| Napropamide | EC_50_ (48h) *D. magna* | 14300000 |
|  | LC_50_ (96h) *C. riparius* | -^2^ |
|  | EC_50_ (7d) *L. gibba* | 240000 |
|  | EC_50_ (72h) *P. subcapitata* | 3400000 |
|  | LC_50_ (96h) *S. gairdneri* | 6600000 |
| Prochloraz | EC_50_ (48h) *D. magna* | 4300000 |
|  | LC_50_ (96h) *C. riparius* | -^2^ |
|  | EC_50_ (7d) *L. gibba* | 171000 |
|  | EC_50_ (72h) *S. subspicatus* | 5500 |
|  | LC_50_ (96h) *O. mykiss* | 1500000 |
| Propioconazol | EC_50_ (48h) *D. magna* | 10200000 |
|  | LC_50_ (96h) *C. riparius* | -^2^ |
|  | EC_50_ (7d) *L. gibba* | 4900000 |
|  | EC_50_ (72h) *N. seminulum* | 93000 |
|  | LC_50_ (96h) *L. xanthurus* | 2600000 |
| Propyzamid | EC_50_ (48h) *D. magna* | 5600000 |
|  | LC_50_ (96h) *C. riparius* | -^2^ |
|  | EC_50_ (7d) *L. gibba* | 1400000 |
|  | EC_50_ (72h) *R. subcapitata* | 2800000 |
|  | LC_50_ (96h) *O. mykiss* | 4700000 |
| Prosulfocarb | EC_50_ (48h) *D. magna* | 510000 |
|  | LC_50_ (96h) *C. riparius* | -^2^ |
|  | EC_50_ (7d) *L. gibba* | 690000 |
|  | EC_50_ (72h) *P. subcapitata* | 49000 |
|  | LC_50_ (96h) *O. mykiss* | 840000 |
| Propioconazol-desthio | EC_50_ (48h) *D. magna* | 10000000 |
|  | LC_50_ (96h) *C. riparius* | -^2^ |
|  | EC_50_ (7d) *L. gibba* | 81000 |
|  | EC_50_ (72h) *S. subspicatus* | 70000 |
|  | LC_50_ (96h) *O. mykiss* | 6630000 |
| Quinmerac | EC_50_ (48h) *D. magna* | 100000000 |
|  | LC_50_ (96h) *C. riparius* | -^2^ |
|  | EC_50_ (7d) *L. gibba* | 96000000 |
|  | EC_50_ (72h) *C. fusca* | 48500000 |
|  | LC_50_ (96h) *O. mykiss* | 86800000 |
| Tebuconazol | EC_50_ (48h) *D. magna* | 2790000 |
|  | LC_50_ (96h) *C. dilutus* | 1899000 |
|  | EC_50_ (7d) *L. gibba* | 144000 |
|  | EC_50_ (72h) *S. subspicatus* | 1960000 |
|  | LC_50_ (96h) *O. mykiss* | 4400000 |
| Terbutryn | EC_50_ (48h) *D. magna* | 2660000 |
|  | LC_50_ (96h) *C. riparius* | -^2^ |
|  | EC_50_ (7d) *L. gibba* | -^2^ |
|  | EC_50_ (72h) *P. subcapitata* | 2400 |
|  | LC_50_ (96h) *O. mykiss* | 1100000 |
| Terbutylazine | EC_50_ (48h) *D. magna* | 21200000 |
|  | LC_50_ (96h) *C. riparius* | -^2^ |
|  | EC_50_ (7d) *L. gibba* | 12800 |
|  | EC_50_ (72h) *P. subcapitata* | 12000 |
|  | LC_50_ (96h) *O. mykiss* | 2200000 |
| Terbutylazine-2-Hydroxy | EC_50_ (48h) *D. magna* | 2800000 |
|  | LC_50_ (96h) *C. riparius* | -^2^ |
|  | EC_50_ (7d) *L. gibba* | -^2^ |
|  | EC_50_ (72h) *P. subcapitata* | 3800000 |
|  | LC_50_ (96h) *O. mykiss* | 2500000 |
| Terbutylazine-desethyl | EC_50_ (48h) *D. magna* | 42000000 |
|  | LC_50_ (96h) *C. riparius* | -^2^ |
|  | EC_50_ (7d) *L. gibba* | -^2^ |
|  | EC_50_ (72h) *P. subcapitata* | 140000 |
|  | LC_50_ (96h) *O. mykiss* | 18000000 |
| Thiacloprid | EC_50_ (48h) *D. magna* | 85100000 |
|  | LC_50_ (96h) *C. dilutus* | 10800 |
|  | EC_50_ (7d) *L. gibba* | 95400000 |
|  | EC_50_ (72h) *S. capricornutum* | 60600000 |
|  | LC_50_ (96h) *O. mykiss* | 90100000 |
| Thiamethoxam | EC_50_ (48h) *D. magna* | 100000000 |
|  | LC_50_ (96h) *C. dilutus* | 55300 |
|  | EC_50_ (7d) *L. gibba* | 90000000 |
|  | EC_50_ (72h) *P. subcapitata* | 100000000 |
|  | LC_50_ (96h) *O. mykiss* | 125000000 |
| Triadimenol | EC_50_ (48h) *D. magna* | 51000000 |
|  | LC_50_ (96h) *C. riparius* | -^2^ |
|  | EC_50_ (7d) *L. gibba* | -^2^ |
|  | EC_50_ (72h) *P. subcapitata* | 9600000 |
|  | LC_50_ (96h) *O. mykiss* | 21300000 |

^1^ Data were obtained from the Pesticide Properties DataBase (PPDB) operated by the University of Hertfordshire ([http://sitem.herts.ac.uk/aeru/iupac/index.htm) (accessed](http://sitem.herts.ac.uk/aeru/iupac/index.htm)(accessed) 5 June 2023).

^2^ No data available.
