# Supplementary material for: Pesticide dynamics in three small agricultural creeks in Hesse, Germany
Source: PeerJ. 2023 Jul 18;11:e15650. doi: 10.7717/peerj.15650 (PMC10361075; doi:10.7717/peerj.15650)
Supplement: Table S9 [file peerj-11-15650-s009.docx]

Table S9: RAC exceedances (yes = 1; no = 0), duration of RAC exceedances (h) and the maximal exceedance factor of detected pesticides.

| Substance | RAC  [ng/L] | Langder Flutgraben | | | | | | | | | | | | | | | | | | |
| --- | --- | --- | --- | --- | --- | --- | --- | --- | --- | --- | --- | --- | --- | --- | --- | --- | --- | --- | --- | --- |
|  |  | 05.15.2018 | | | | | 05.24.2018 | | | | | | | 05.31.2018 | | | | | | |
|  |  | RAC exceedance | Duration [h] | Max. concentration [ng/L] | | Max. exceedance factor | RAC exceedance | Duration [h] | Max. concentration [ng/L] | | Max. exceedance factor | | | RAC exceedance | Duration [h] | | | Max. concentration [ng/L] | | Max. exceedance factor |
| Diflufenican | 25 | 1 | 2 | 32.0 | | 1.28 | 1 | 3 | 40.1 | | 1.60 | | | 1 | 5 | | | 46.6 | | 1.86 |
| (S)-Metolachlor | 1220 | 1 | 18 | 8,260 | | 6.69 | 1 | 24 | 14,500 | | 11.9 | | | 1 | 18 | | | 5,170 | | 4.24 |
| Prosulfocarb | 3800 | 1 | 3 | 9,150 | | 2.41 | 1 | 12 | 12,300 | | 3.24 | | | 1 | 5 | | | 5,250 | | 1.38 |
| Terbutylazine | 1900 | 1 | 2 | 3,090 | | 1.63 | 1 | 18 | 8,690 | | 4.58 | | | 1 | 18 | | | 4,920 | | 2.59 |
| Thiacloprid | 4 | 1 | 24 | 25.8 | | 6.45 | 1 | 18 | 10.5 | | 2.63 | | | 1 | 8 | | | 6.40 | | 1.60 |
|  |  | Waschbach | | | | | | | | | | | | | | | | | | |
|  |  | 05.20.2017 | | | | | 05.31.2017 | | | | | | | 06.04.2017 | | | | | | |
|  |  | RAC exceedance | Duration [h] | Max. concentration [ng/L] | | Max. exceedance factor | RAC exceedance | Duration [h] | Max. concentration [ng/L] | | Max. exceedance factor | | | RAC exceedance | Duration [h] | | | Max. concentration [ng/L] | | Max. exceedance factor |
| Clothianidin |  | 0 | - | - | | - | 0 | - | - | | - | | | 1 | 17 | | | 97.8 | | 14.0 |
| Flufenacet |  | 0 | - | - | | - | 0 | - | - | | - | | | 1 | 6 | | | 889 | | 2.49 |
| Imidacloprid |  | 0 | - | - | | - | 0 | - | - | | - | | | 1 | 12 | | | 37.3 | | 4.14 |
| Metamitron |  | 0 | - | - | | - | 1 | 4 | 78,228 | | 2.06 | | | 1 | 7 | | | 58,209 | | 1.53 |
| Metazachlor |  | 0 | - | - | | - | 1 | 10 | 6,762 | | 5.4 | | | 0 | - | | | - | | - |
| (S)-Metolachlor |  | 0 | - | - | | - | 0 | - | - | | - | | | 1 | 5 | | | 3,893 | | 3.19 |
| Prosulfocarb |  | 0 | - | - | | - | 0 | - | - | | - | | | 1 | 1 | | | 3,886 | | 1.02 |
| Thiacloprid |  | 1 | 13 | 5.76 | | 1.44 | 1 | 1 | 4.19 | | 1.05 | | | 1 | 2 | | | 4.60 | | 1.15 |
| Thiamethoxam |  | 0 | - | - | | - | 0 | - | - | | - | | | 1 | 6 | | | 97.7 | | 2.27 |
|  |  | Waschbach | | | | | | | | | | | | | | | | | | |
|  |  | 03.30.2018 | | | | | 05.15.2018 | | | | | | 05.24.2018 | | | | | | | |
|  |  | RAC exceedance | Duration [h] | Max. concentration [ng/L] | Max. exceedance factor | | RAC exceedance | Duration [h] | | Max. concentration [ng/L] | | Max. exceedance factor | RAC exceedance | | | Duration [h] | Max. concentration [ng/L] | | Max. exceedance factor | |
| Clothianidin | 7 | 1 | 3 | 8.20 | 1.17 | | 1 | 3 | | 11.4 | | 1.63 | 0 | | | - | - | | - | |
| Thiacloprid | 4 | 0 | - | - | - | | 1 | 21 | | 17.4 | | 4.35 | 1 | | | 24 | 705 | | 176 | |
| Thiamethoxam | 43 | 0 | - | - | - | | 1 | 3 | | 85.5 | | 1.99 | 0 | | | - | - | | - | |
|  |  | Waschbach | | | | | Weidgraben | | | | | |  | | | | | | | |
|  |  | 06.14.2018 | | | | | 15.05 | | | | | |  | | | | | | | |
|  |  | RAC exceedance | Duration [h] | Max. concentration [ng/L] | Max. exceedance factor | | RAC exceedance | Duration [h] | | Max. concentration [ng/L] | | Max. exceedance factor |  | | |  |  | |  | |
| Thiacloprid | 4 | 1 | 18 | 15.2 | 3.80 | | 1 | 3 | | 4.2 | | 1.05 |  | | |  |  | |  | |
| Thiamethoxam | 43 | 0 | - | - | - | | 1 | 22 | | 92.2 | | 2.14 |  | | |  |  | |  | |
