# Supplementary figures and images for: Pesticide dynamics in three small agricultural creeks in Hesse, Germany
Source: PeerJ. 2023 Jul 18;11:e15650. doi: 10.7717/peerj.15650 (PMC10361075; doi:10.7717/peerj.15650)

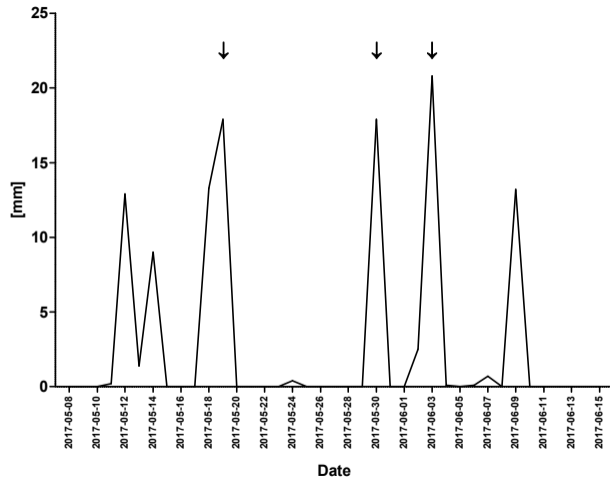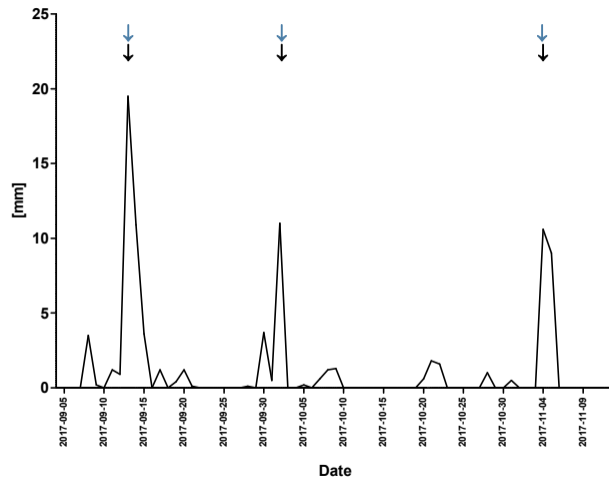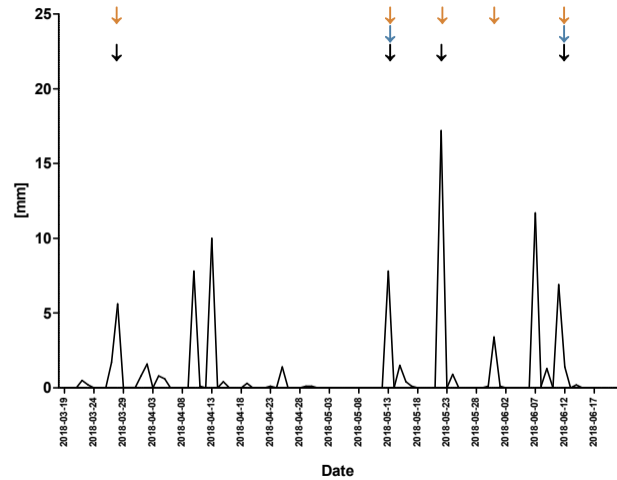

Supplement: Figure S1 — Total precipitation [mm] based on data from the Hessian State Agency for Nature Conservation, Environment and Geology (https://www.hlnug.de/static/pegel/wiskiweb3/webpublic/#/overview/Niederschlag6?filter=%7B%7D), measured at the Niddatal-Nieder-Florstadt station. The arrows symbolize the individual sampling campaigns for the different creeks (black arrows: Waschbach; blue arrows: Weidgraben; brown arrows: Langder Flutgraben). [file peerj-11-15650-s010.pdf]

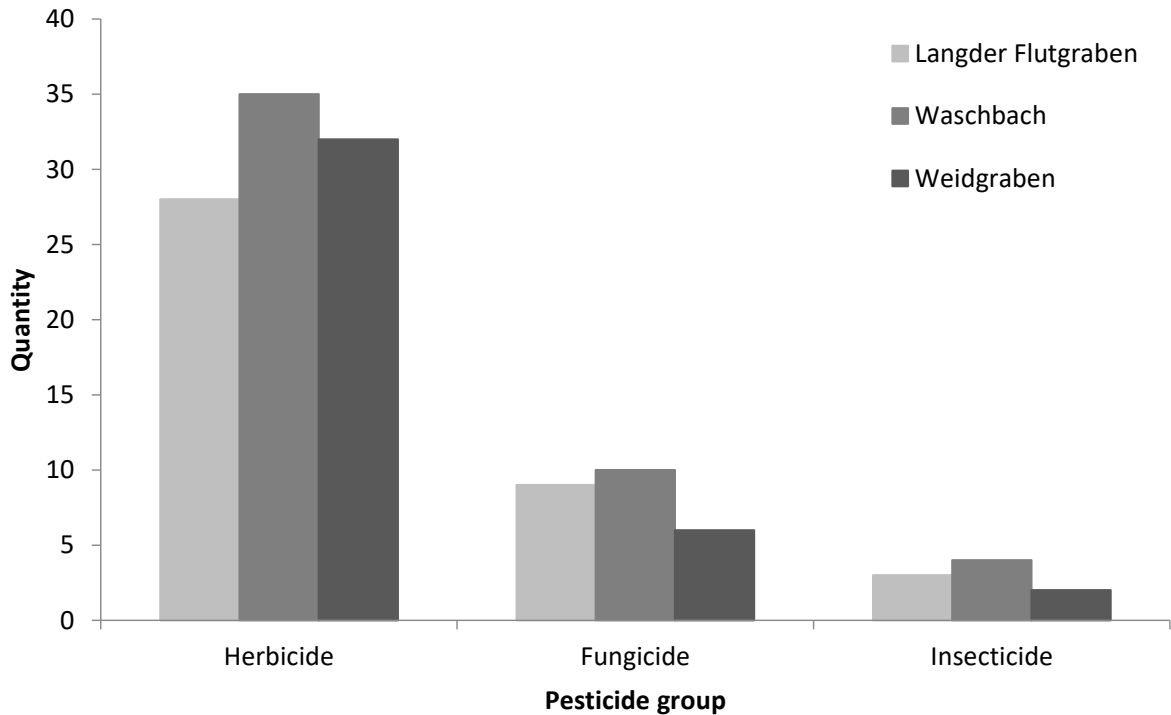

Supplement: Figure S2 [file peerj-11-15650-s011.pdf]

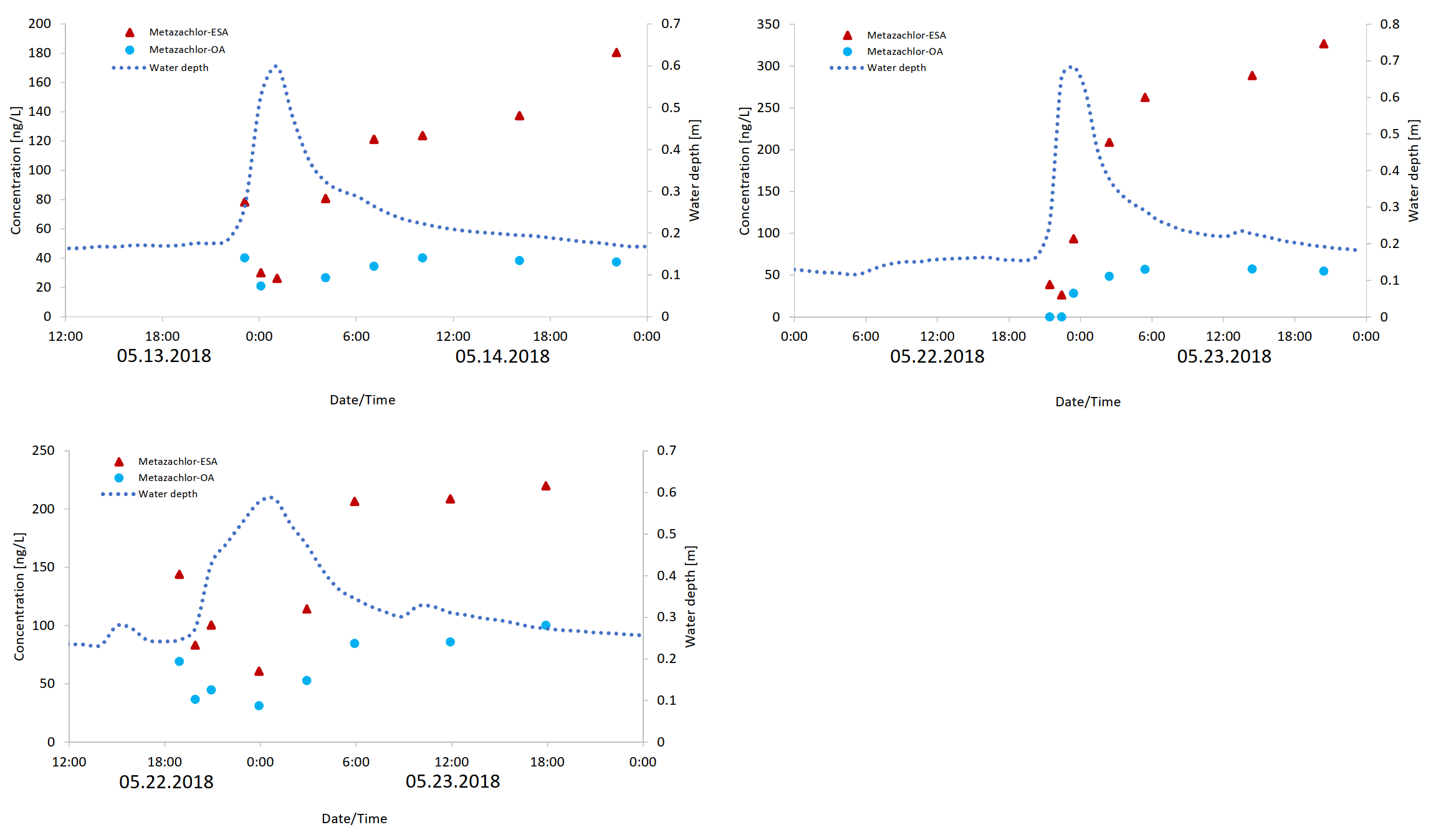

Supplement: Figure S3 — Measured concentrations of the transformation products metazachlor-ESA and metazachlor-OA at Langder Flutgraben at two sampling events (panel A: 05/13 - 05/14/2018; panel B: 05/22 –05/23/2018) and at Waschbach at a sampling event (panel C: 05/22/2018 –05/23/2018) with the corresponding water depths. [file peerj-11-15650-s012.png]
